# Inhibition of USP30 Promotes Mitophagy by Regulating Ubiquitination of MFN2 by Parkin to Attenuate Early Brain Injury After SAH

**DOI:** 10.1007/s12975-023-01228-3

**Published:** 2023-12-26

**Authors:** Yang Liu, Chenbei Yao, Bin Sheng, Simin Zhi, Xiangxin Chen, Pengfei Ding, Jiatong Zhang, Zhennan Tao, Wei Li, Zong Zhuang, Jiannan Mao, Zheng Peng, Huiying Yan, Wei Jin

**Affiliations:** 1 Department of Neurosurgery, Nanjing Drum Tower Hospital Clinical College of Nanjing University of Chinese Medicine, Nanjing, 210008 Jiangsu China; 2 Department of Neurosurgery, Nanjing Drum Tower Hospital, Affiliated Hospital of Medical School, Nanjing University, Nanjing, 210008 Jiangsu China; 3 Department of Neurosurgery, Nanjing Drum Tower Hospital Clinical College of Nanjing Medical University, Nanjing, 210008 Jiangsu China

**Keywords:** Ubiquitin-specific protease 30 (USP30), Mitochondrial fusion protein 2 (MFN2), Subarachnoid hemorrhage (SAH), Mitophagy, Ubiquitination, MF094

## Abstract

Subarachnoid hemorrhage (SAH) is a type of stroke with a high disability and mortality rate. Apoptosis caused by massive damage to mitochondria in neuron cells and inflammatory responses caused by high extracellular ATP lead to poor outcomes. USP30 is a deubiquitinating enzyme that inhibits mitophagy, resulting in a failure to remove damaged mitochondria in a timely manner after SAH; nevertheless, the pathway through which USP30 inhibits mitophagy is unknown. This study evaluated the neuroprotective role and possible molecular basis by which inhibiting USP30 to attenuate SAH-induced EBI by promoting neuronal mitophagy. We used an in vitro model of hemoglobin exposure and an in vivo model of intravascular perforation. Increased expression of USP30 was found after SAH in vivo and in vitro, and USP30 inhibition expression in SAH mice treated with MF094 resulted in significant improvement of neurological injury and inflammatory response and mediated good outcomes, suggesting a neuroprotective effect of USP30 inhibition. In cultured neurons, inhibition of USP30 promoted ubiquitination modification of mitochondrial fusion protein 2 (MFN2) by E3 ubiquitin ligase (Parkin), separating damaged mitochondria from the healthy mitochondrial network and prompting mitophagy, causing early clearance of damaged intracellular mitochondria, and reducing the onset of apoptosis. The high extracellular ATP environment was meliorated, reversing the conversion of microglia to a pro-inflammatory phenotype and reducing inflammatory injury. USP30 inhibition had no autophagy-promoting effect on structurally and functionally sound mitochondria and did not inhibit normal intracellular ATP production. The findings suggest that USP30 inhibition has a neuroprotective effect after SAH by promoting early mitophagy after SAH to clear damaged mitochondria.

## Introduction

Subarachnoid hemorrhage (SAH) is a severe cerebrovascular disease that significantly affects human health [1]. Although it accounts for only 5 to 10% of all stroke cases, its mortality rate is very high. Approximately 50% of patients are left with disabilities, severely impacting their quality of life. In recent years, the role of mitochondria in traumatic diseases has gained attention [2]. Mitochondria are known as the powerhouses of cells, because they are essential for energy production, calcium ion (Ca2 +) storage, and the regulation of cell proliferation and metabolism [3]. SAH leads to mitochondrial damage, which subsequently triggering a cascade of reactions such as oxidative stress, energy imbalance, and inflammatory responses [2, 4]. This cascade results in phenomena such as apoptosis and high levels of damage molecules such as adenosine triphosphate (ATP) released from damaged cells, which gives rise to early brain injury (EBI) and neuroinflammation [5]. The early clearance of damaged mitochondria is an approach to treat SAH and improve outcomes [2, 6].

Mitophagy is the optimal pathway for early clearance of damaged mitochondria [7, 8], effectively reducing cell apoptosis after SAH. Our focus is on a deubiquitinase (DUB) called USP30, found on the outer mitochondrial membrane (OMM) and a member of the USP subfamily[9]. It is a critical factor in regulating mitophagy [10, 11]. After mitochondrial damage occurs, USP30 expression in OMM increases rapidly and counteracts mitophagy by removing the ubiquitin tag placed by E3 ubiquitin ligase (Parkin), resulting in the accumulation of mitochondrial fusion protein (MFN2) [12]. These findings suggest that reducing USP30 expression after the onset of mitochondrial damage using the USP30-specific inhibitor MF094 promotes mitophagy, clears damaged mitochondria, and inhibits cell apoptosis [13]. This mechanism might be exploited to treat early EBI and improve outcomes after SAH.

ATP is a damage molecule released after SAH, and neuronal mitochondrial damage is one of its essential sources. Substantial amounts of ATP are released extracellularly after SAH, activating an essential purinergic receptor on the surface of microglia in the CNS, the P2X purinergic receptor 7 (P2X7); its expression is positively correlated with the extracellular ATP concentration [14]. The high expression of P2X7 causes microglia to transform from a resting state to a predominantly pro-inflammatory M1 type, which is involved in several physiopathological processes, including the activation of NLR family pyridine domain containing 3 (NLRP3) inflammatory vesicles on macrophages/microglia and the release of inflammatory cytokines and chemokines, resulting in the release of IL-6,IL-1β, TNF-α, and other inflammatory factors, causing severe neuroinflammatory responses [15, 16]. Inhibition of P2X7 expression can shift microglia toward the M2 type, which is anti-inflammatory, releasing various anti-inflammatory factors, including IL-10, IL-4, and TNF-β, and initiating neural repair [17]. Mitophagy can reduce microglia-mediated inflammatory responses by selectively removing damaged mitochondria to reducing extracellular ATP levels and inhibiting P2X7 activation [18].

Therefore, we studied the removal of damaged mitochondria after SAH by inhibiting USP30 and increasing the ubiquitination of MFN2 by Parkin to promote mitophagy. The aim was to reduce apoptosis, decrease extracellular ATP, reduce the neuroinflammatory responses, and improve outcomes.

## Materials and Methods

### Collection of Cerebrospinal Fluid (CSF)

CSF samples were obtained from patients with intracerebral herniation decompression, and samples from the control group were obtained intraoperatively from patients with facial myoclonus. Samples from both groups were stored under liquid nitrogen for rapid freezing without contamination.

### Animal and SAH Models

Eighty-seven healthy adult male mice C57BL/6 J (6–8 weeks, 18–20 g, Huachuang Sino, China) were randomly divided into N groups with post hoc power analysis to verify sample size (*α* = 0.05, adequate statistical power of 80%). Sixty healthy female C57BL/6 J pups (0–24 h, 1.5–2.0 g, Huachuang Sino) and 25 healthy adult pregnant C57BL/6 J rats (16–17 days of pregnancy, 40–50 g, Huachuang Sino) were used to extract primary microglia and primary neurons, respectively. All animals were housed in alternating light/dark (12 h of light/dark each) environments at constant temperature (24 ± 2 °C) and humidity (65 ± 5%), with free access to water and food and free from pathogens.

The SAH model in mice was induced by intravascular puncture [19], and mice were anesthetized with 3% isoflurane (RWD, #R510-22, China) and placed on an operating table, during which 1.5% isoflurane was maintained by continuous inhalation. The external carotid artery (ECA) and internal carotid artery (ICA) of mice were fully exposed, and the ECA was ligated and dissected distally to the bifurcation, the vessel was severed close to the disconnected end. A pre-prepared bolus wire (CinoTech, # A2-162,350, China) was inserted backward through the internal carotid artery from the ECA incision into the middle cerebral artery bifurcation, and after a slight breakthrough sensation, the bolus wire was retrieved. The procedure was identical except for the punctured vessel in the sham-operated group. The breach was then ligated to prevent bleeding. After surgery, 1 mL of saline was injected intraperitoneally, and the animals were transferred to a heating blanket to await resuscitation, after which they were transferred to a specialized care room where jelly and water were provided to replenish their energy [20]. Neurological function was assessed 24 h postoperatively using a modified Garcia score, excluding mice with scores ≤ 6 or ≥ 15 scores.

### Lateral Ventricular Injection Drug Delivery

MF094 (#HY-112438, MCE, USA) was dissolved with 5% dimethyl sulfoxide (DMSO, #D4540, Sigma, USA) and diluted with saline. Mice were anesthetized with isoflurane 3 days before induction of SAH as described above. The animals were fixed on a brain stereotactic apparatus, and the skin of the head was disinfected and incised along the midline to expose the anterior hallux point (bregma). The coordinates of lateral ventricular localization were 0.6 mm posterior to the bregma with a parasternal opening of 1.5 mm (left parasternal opening) and a depth of 1.7 mm. After localization, a 0.88-mm diameter drill was used to bury the cannula, and the outer end of the cannula was connected to a 15-cm PE tube to determine if the burying was successful [21]. Then, the drug was pumped into the lateral ventricles through the PE tube at 0.5 µL/min using a 10-µL microinjector. After a successful injection, the scalp was sutured, and the cannula was left in place. The mice were subsequently cared for as after the SAH model was created. After that, the drug was injected daily until the end of the experiment. The unloaded group was operated as the experimental group, except that MF094 was replaced with equal saline.

### Animal Experiments

Animal experiments were designed in two parts. In the first part, 36 mice were randomly divided into four groups according to the drug’s instructions: sham (*n* = 6), SAH + MF094 (1 mg/kg, *n* = 10), SAH + MF094 (5 mg/kg, *n* = 10), and SAH + MF094 (10 mg/kg, *n* = 10). Neurological tests were performed after excluding the non-compliant mice, and 5 mg/kg was selected as the best dose for the subsequent experiments. In the second part, 51 mice were randomly divided into four groups: sham (*n* = 6), SAH (*n* = 15), SAH + MF094 (5 mg/kg, *n* = 15), and SAH + Vehicle (*n* = 15), and modified Garcia scores were recorded after excluding non-compliant mice at one and three days postoperatively. Three days later, mice were euthanized, and brain tissues were removed for subsequent experiments.

### Primary Neuron Culture and InVitro Models

The medium was prepared as follows: Neurobasal Medium (#21,103–049, Gibco, USA) containing 2% B-27 Supplement (#A3582801, Gibco), 1% GlutaMAX Supplement (#35,050,079, Gibco) and 1% penicillin–streptomycin (#10,378,016, Gibco). Pregnant mice were sterilized and euthanized at about 16–17 days of gestation, and the fetal sacs were removed. After careful rupture of the fetal sac, brain tissue was taken, and the meninges were removed; all brains were cut into segments of approximately 1 mm^3^; then, TrypLE (# 12,563,029, Gibco, USA) was added and digested at 37 °C for 10 min. We added 2 mL of fetal bovine serum to terminate the digestion. Tissue was pipetted ten times, filtered through a 70-μm filter (# 352,350, Falcon, USA), and repeated 3–4 times. The filtered cells were centrifuged at 1000 rpm for 5 min. After centrifugation, the supernatants were discarded, and the cells were resuspended in a culture medium and transferred to Petri dishes covered with poly-lysine (Pold-D-lysine, #ST508, Beyotime, China) [22]. The primary neurons matured after half-transfer on days 3 and 5 and full transfer on day 7.

Cells were cultured in an incubator at 37 °C and 5% CO^2^ for more than 7 days, and bovine hemoglobin (Sigma, USA) was dissolved in a culture medium (25 µmol/L) to establish the SAH model in vitro. MF094 was diluted to 180 nmol/L with culture medium and added to the medium 24 h in advance [23].

### Primary Microglia Culture andIn VitroModels

The medium was prepared as follows: high-sugar Dulbecco’s modified Eagle medium (# C11995500BT, Gibco) containing 10% fetal bovine serum (#10099141C, Gibco) and 1% penicillin–streptomycin (#10,378,016, Gibco). We selected the brain tissue of young rats within 24 h of birth. The extraction procedure is the same as for neurons. After collecting the cells, changing the medium on days 3 and 7, and maturing at approximately day 10, the suspended microglia were collected and transferred to culture dishes for subsequent experiments [24].

Cells were cultured at 37 °C and 5% CO_2_ for more than 10 days, and ATP (#D7378, Beyotime) was added to the culture medium at various concentrations to create in vitro models according to experimental needs. Because ATP is unstable, it was re-added every 12 h.

### Extraction of Cytoplasmic and Nuclear Proteins

Using the Cytoplasmic and Nuclear Protein Extraction Kit (#KTP3001, Abbkine, USA), the corresponding proteins were extracted by adding the corresponding reagents according to the manufacturer’s instructions.

### Measurement of Brain Water Content

The mice were sacrificed by over-inhalation of isoflurane, and the brain tissues were measured for wet weight, then dried in an oven at 70 °C for 24 h. The brain tissues were removed and measured again to calculate the water loss.

### Western Blot (WB)

Twenty milligrams of brain tissue from the temporal lobe of the injured hemisphere were collected and cut into pieces. Cells were collected directly and centrifuged. Cultured cells or brain tissue were lysed on ice with a mixture of RIPA (#89,901, Thermofisher, USA), 1% protease inhibitor (#C0001, TargetMol, USA), and 0.1% super nuclease (#D7121-5KU, Beyotime). Protein quantification was performed using the Dual Chondroitin Protein Assay Kit (#P0009, Beyotime). The same mass of proteins was loaded onto sodium dodecyl polyacrylamide gels (#PG111, Epizyme, China) before transferring to polyvinylidene fluoride membranes (#IPVH00010, Sigma, USA). The membranes were blocked with 5% skim milk powder for 1 h at room temperature and incubated with primary antibodies overnight at 4 °C, followed by incubation with corresponding secondary antibodies for 1 h at room temperature. We used a standard electrochemiluminescence substrate (#BMU102-CN, Abbkine, USA), incubated the blots with the working solution for 1–5 min, and analyzed the bands using ImageJ. Antibodies used in WB are listed in Table 1.

**Table 1:** Antibodies and primer sequences involved

Antibody	USP30	Ablconal (#A12862)
Antibody	PINK1	Santa Cruz (#sc-517353)
Antibody	PARKIN	Santa Cruz (#sc-32282)
Antibody	MFN2	Proteintech (#12,186–1-AP)
Antibody	Cytochrome C	Proteintech (#66,264–1-Ig)
Antibody	AIF	Ablconal (#A19536)
Antibody	LC3	Proteintech (#14,600–1-AP)
Antibody	Ubiquitin	Santa Cruz (#sc-8017)
Antibody	P2X7	Proteintech (#28,207–1-AP)
Antibody	NLRP3	Proteintech (#68,102–1-Ig)
Antibody	CD86	Santa Cruz (#sc-28347)
Antibody	CD206	Santa Cruz (#sc-376108)
Antibody	CD206	Proteintech(#18,704–1-AP)
Antibody	NeuN	Cell Signaling (#24307S)
Antibody	Iba-1	Abcam (#ab5076)
Antibody	β-Actin	Cell Signaling (#3700S)
Antibody	β-Tubulin	Cell Signaling (#2128S)
qPCR	USP30-F	AGTCACTTGCCACACGAGAG
qPCR	USP30-R	CCCAAGTGGCAGCTGGAATA
qPCR	MFN2-F	GTGGGCTGGAGACTCATCG
qPCR	MFN2-R	CTCACTGGCGTATTCCGCAA
qPCR	CD86-F	GGTGGCCTTTTTGACACTCTC
qPCR	CD86-R	TGAGGTAGAGGTAGGAGGATCTT
qPCR	CD206-F	CTCTGTTCAGCTATTGGACGC
qPCR	CD206-R	CGGAATTTCTGGGATTCAGCTTC
qPCR	IL-6-F	CCAAGAGGTGAGTGCTTCCC
qPCR	IL-6-R	CTGTTGTTCAGACTCTCTCCCT
qPCR	IL-4-F	GGTCTCAACCCCCAGCTAGT
qPCR	IL-4-R	GCCGATGATCTCTCTCAAGTGAT
qPCR	IL-10-F	GCTCTTACTGACTGGCATGAG
qPCR	IL-10-R	CGCAGCTCTAGGAGCATGTG
qPCR	IL-1β-F	TTCAGGCAGGCAGTATCACTC
qPCR	IL-1β-R	GAAGGTCCACGGGAAAGACAC
qPCR	TNF-α-F	GACGTGGAACTGGCAGAAGAG
qPCR	TNF-α-R	TTGGTGGTTTGTGAGTGTGAG
qPCR	TNF-β-F	CCACCTCTTGAGGGTGCTTG
qPCR	TNF-β-R	CATGTCGGAGAAAGGCACGAT
qPCR	GAPDH-F	AGGTCGGTGTGAACGGATTTG
qPCR	GAPDH-R	TGTAGACCATGTAGTTGAGGTCA

### Real-Time PCR (qPCR)

TRIzol reagent (#R401-01, Vazyme, China) was used to extract mRNA according to the manufacturer's instructions. Reverse transcription reagent (#R323-01, Vazyme) was mixed with mRNA to remove and reverse transcribe it into cDNA. qPCR was performed using SYBR Greenmix (#Q331-AA, Vazyme) using a PCR system (Applied Biosystems, USA) was performed. Results were analyzed using the 2-ΔΔCt method. Primers used in qPCR are listed in Table 1.

### Flow Cytometry (FC)

After collecting cells, we added DCFH-DA (#S0033S, Beyotime), Mito-Tracker (#C1032, Beyotime), and other related reagents. Cells were incubated for 15 min at room temperature, protected from light. Detection was performed using C6 FC. If the addition of antibodies is required, the cells were collected and fixed with 4% Fixative Solution (#BL539A, Bioshard, China) for 15 min, as both CD86 and CD206 are cell surface antigens and do not require additional permeabilize. After blocking with QuickBlock (#P0260, Beyotime) for 15 min, high concentrations of targeting primary antibodies from different species sources were added and incubated in a mixture at room temperature for 3 h. After three washes with phosphate-buffered saline (PBS), fluorescent secondary antibodies corresponding to the source of primary antibodies were added. We incubate at room temperature for 1 h and wash three times with PBS and perform the assay. The results were analyzed using FlowJo (version 10). Antibodies used in FC are listed in Table 1.

### Immunofluorescence (IF)

Intact mouse brain tissue was removed and placed in 4% Fixative Solution for 24 h and dehydrated with a sucrose gradient (15% and 30%). Frozen sections of 10 μm were blocked with 1% bovine albumin serum for 1 h at 25 °C. The targeting primary antibody was then added and incubated overnight at 4 °C. After three washes with PBS, a fluorescent secondary antibody corresponding to the primary antibody source was added, incubated for 1 h at room temperature against light, and washed three times with PBS and finally DAPI containing a fluorescent bursting agent (#P0131, Beyotime). If cells were used, the same operation was performed after fixation, membrane lysis, and blockade in 24-well plates. Fluorescence images were obtained using a Leica Thunder fluorescence microscope system and an Olympus FV3000 confocal microscope system. Image analysis was performed using Olympus CellSens software and ImageJ software. Antibodies used in WB are listed in Table 1.

### Cell-Counting Kit 8 (CCK8) Assay

The medium containing 10% CCK8 was added to the cells and incubated at 37 °C for 30–60 min, and an absorbance assay was performed at 450 nm.

### ATP Measurement

CSF or medium was mixed with the relevant reagents according to the manufacturer’s instructions using the ATP Content Assay Kit (#BC0300, Solarbio, USA). The supernatants were placed in cuvettes, and the absorbance value was measured at 340 nm with a UV spectrophotometer to calculate the ATP concentration according to a standard formula.

### Immunoprecipitation (CO-IP)

Using an immunoprecipitation kit (#PK10007, Proteintech, China) according to the manufacturer’s instructions, we centrifuged the cells, extracted the proteins, and added specific antibody A. Cells were incubated overnight at 4 °C with rotation. The negative control group was treated with the same amount of control immunoglobulin of the same genus, and the subsequent operation was the same as that of the experimental group. Subsequently, rProtein A/G beads slurry was added to precipitate the immune complexes, eluted, and boiled for 5 min. A subsequent analysis was performed by WB.

### Nissl Staining

A Nissl staining kit (#G1432, Solarbio, China) was used to fix frozen sections of 10 µM with Carnoy fixative, stained with methyl violet staining solution for 10–20 min, rinsed three times with PBS, fractionated with Nissler fractionation solution for 4–8 s, made transparent in xylene, and sealed with neutral resin according to the manufacturer’s instructions.

### TdT-Mediated dUTP Nick End Labeling (TUNEL) Staining

A TUNEL Staining Kit (#C1086, Beyotime) was used. After fixation, permeabilization, and closure of the cells and frozen sections according to the manufacturer’s instructions, 50 µL of TUNEL assay solution was prepared, and 1:9 TdT enzyme: fluorescent labeling solution was added and incubated for 60 min at 37 °C and protected from light. After three washes in PBS, the cells were observed by fluorescence microscopy at 480 nm.

### Transmission Electron Microscopy

Cells were collected by centrifugation followed by 2.5% glutaraldehyde (#30,092,436, Sinopharm, China) before fixation for 4 h and rinsed three times in PBS for 15 min each. The cells were fixed with 1% osmium acid for 2 h at room temperature and then rinsed three times with PBS for 15 min each time. Cells were then dehydrated in alcohol (#100,092,683, Sinopharm, China) in gradients (30%, 50%, 70%, 80%, 85%, 90%, and 100%) for 15–20 min each time. The samples were then permeabilized with acetone: epoxy resin (2:1) at 37 °C for 8–12 h. The permeabilized samples were placed in an embedding plate and polymerized with the embedding agent epoxy resin (#90,529–77-4, SPI, China) at 60 °C for 48 h. Finally, the cells were cut into 80–100-nm sections, double-stained, and observed by electron microscopy (#TECNAI G 20 TWIN, FEI USA).

### Detection of Differentially Expressed Genes

Raw data were obtained from the GEO database (GSE207496). Cells from Parkin-overexpressing and Parkin-knockout mice were analyzed separately. We used *P* ≤ 0.05 (indicated by vertical coordinate–log10 (*P* value)), fold-change ≥ 2, or <  − 2 (indicated by log2 (fold-change) in horizontal coordinates). Differential gene expression was analyzed, and heat maps were plotted using Sento Academic Tools (https://www.xiantaozi.com). Each point represented a differentially expressed gene. The size and color of the dots can indicate additional properties, with the color of the dot marking the corresponding gene as upregulated (red), downregulated (blue), and non-differential (black).

### Reciprocal Sequence and Molecular Docking Model

The lysine site of Parkin acting on Mfn2 was predicted using the Ubinet 2.0 tool, with Mfn2 as the substrate and the corresponding motif. The lysine site was predicted by downloading the protein (Parkin) structure file (PDB: AF-O60260-F1) from the Protein Data Bank and the protein (MFN2) structure file (PDB: AF O95140-F1). The HDOCK Server was used for flexible docking to generate 1000 different conformational orientations. This server was also used to obtain the electrostatic and van der Waals interactions between the object and the subject, from which the Docking Score was calculated. The best scoring conformations were obtained by ranking the docking scores. Python (Version 3.7.7)-Pymol (Version 2.4.0) was used for docking graphical processing and intermolecular spatial distances (distances less than 5 Å were considered as having significant interactions), and the final published images were generated by removing intramolecular water molecules, color-coding of protein subunits, and visualization.

### Enzyme-Linked Immunosorbent Assay (ELISA)

For the detection of USP30 in the patient’s CSF, we chose Human USP30 ELISA KIT (#YJ002365, Mlbio,China), which has a detection range of 0.1–1600 pg/mL. Our pre-test results indicate that an additional fivefold dilution is required to perform the assay. Prepare reagents, samples, and standards as instructed. Add 10μL of sample to be tested and 40μL of sample dilution into each well, add 100μL of horseradish peroxidase (HRP)-labeled detection antibody into each of the standard and sample wells, and incubate at 37 ℃ for 1 h. Discard the liquid, fill up each well with the washing solution, and leave it to stand for 1 min, then discard the washing solution, and repeat the washing of the plate for 5 times. Next, add 50μL each of substrate A and B to each well and incubate at 37 ℃ for 15 min. Add 50 μL stop solution to each well and read at 450 nm within 5 min.

### Statistical Analysis

All data were statistically analyzed using Prism 8.02 (GraphPad Software, USA), SPSS 22.0 (SPSS Inc., USA), and MedCalc 20 (MedCalc Software, Mariakirk, Belgium). Values were expressed as mean ± standard deviation. All data were first tested for normality and the chi-square test. Categorical variables were expressed as frequencies and percentages, and group comparisons were made using the *χ*^2^ or Fisher test. The two-tailed Student’s *t*-test was used to assess differences between two groups, and if chi-squared was satisfied, a one-way analysis of variance (ANOVA) with more than two groups was used, followed by Tukey’s test. If the chi-square was not satisfied, one-way ANOVA with more than two groups was used for comparison using the Kruskal–Wallis one-way ANOVA. Differences where *P* < 0.05 were considered statistically significant.

## Result

### After SAH, USP30 Levels and ATP Levels in CSF Can Predict the Severity of Injury and Prognosis of Patients

**Fig. 1:**
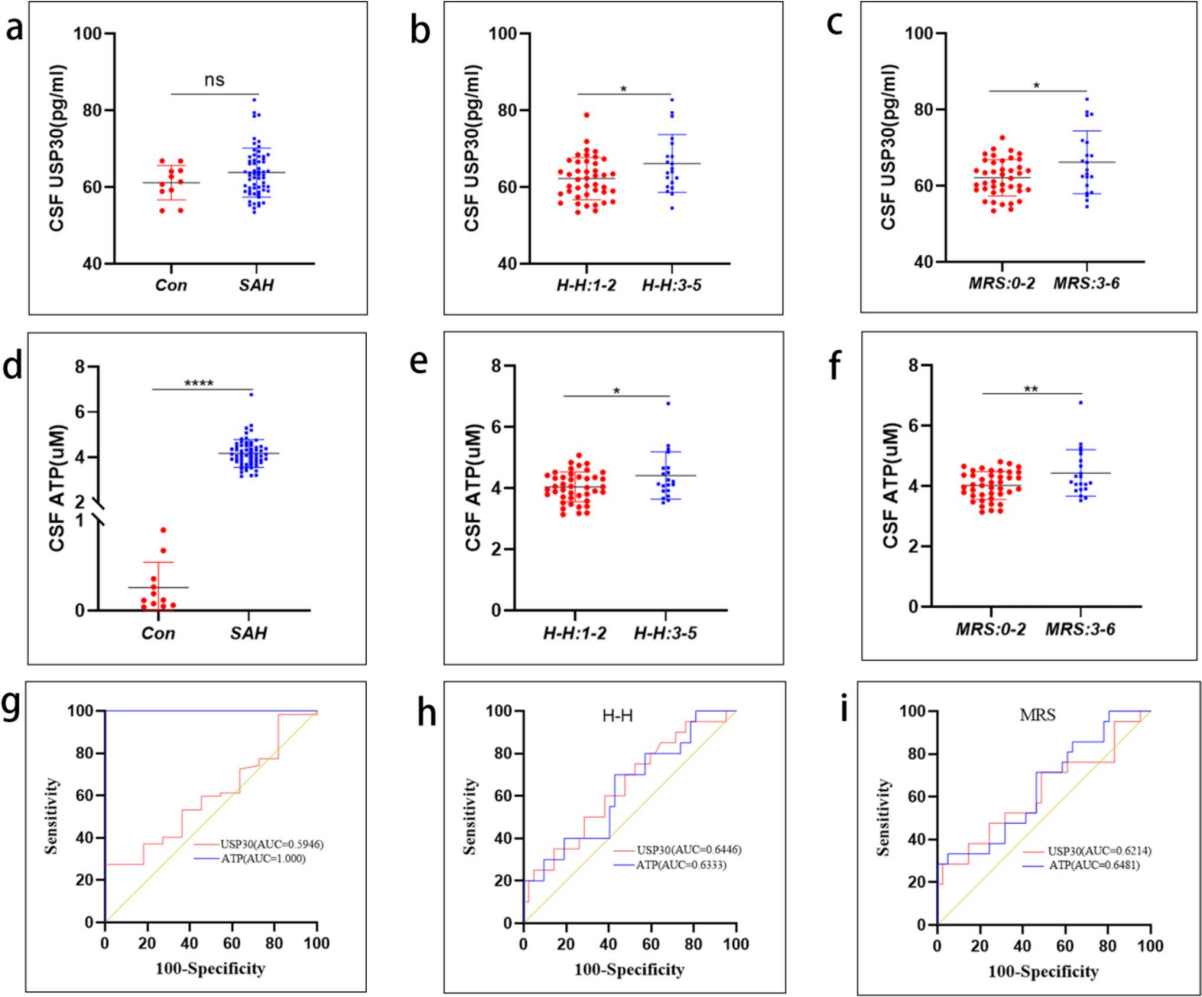
Predictive role of USP30 levels and ATP levels in the CSF of SAH patients. **a** USP30 levels in the CSF of patients in the control and SAH groups (Con: *n* = 11, SAH: *n* = 62, *t*-test). **b** Correlation analysis of USP30 levels with Hunt-Hess scores in the SAH group (H–H 0–2: *n* = 42, H–H 3–5: *n* = 20, *t*-test). **c** Correlation analysis between USP30 levels and MRS scores in the SAH group (MRS 0–2: *n* = 41, MRS 3–5: *n* = 21, *t*-test). **d** ATP levels in CSF of patients in the control and SAH groups (Con: *n* = 11; SAH: *n* = 62, *t*-test. **e** Correlation analysis of ATP levels with Hunt-Hess scores in the SAH group (H–H 0–2: *n* = 42, H–H 3–5: *n* = 20, *t*-test). **f** Correlation analysis between ATP levels and MRS scores in the SAH group (MRS 0–2: *n* = 41, MRS 3–5: *n* = 21, *t*-test). **g** ROC curves of CSF in the control and SAH groups. **h** ROC curves for H–H scores in SAH group. **i** ROC curves for MRS scores in SAH group. ns: not significant, *P < 0.05, **P < 0.01, ***P < 0.001, and ****P < 0.0001

Patients’ characteristics are listed in Table 2. Compared with the control group, USP30 levels in the CSF of SAH patients were elevated on days 0–3 after bleeding (Con vs SAH, 61.20 ± 4.47 pg/mL vs. 63.80 ± 6.36 pg/mL), but were not statistically significant (Fig. 1a), considering that there were too few data in the Con group, resulting in insignificant differences in the results. Hunt-Hess (H–H) score results showed that USP30 levels in the SAH group predicted the severity of injury in patients (H–H 0–2 vs. H–H 3–5, 62.27 ± 5.48 pg/mL vs. 66.20 ± 7.52 pg/mL) (Fig. 1b). The study also showed that USP30 levels in the SAH group were positively correlated with Modified Rankin Scale (MRS) (MRS 0–2 vs MRS 3–6, 62.15 ± 4.80 pg/mL vs. 66.24 ± 8.25 pg/mL) (Fig. 1c), suggesting that USP30 in CSF can predict patient prognosis.

**Table 2:** Univariate analysis comparing patients with poor and good outcomes

Variable	MRS: 0–2	MRS: 3–6	*P* value
Males/females	16 (26%)/25 (40%)	12 (19)/9 (15%)	0.1805
Mean age in years	57 (34–69)	58 (43–78)	0.7840
Hypertension	23 (37%)	10 (16%)	0.7851
DSA			0.1435
Positive	35 (56%)	21 (34%)	
Negative	6 (10%)	0	
Aneurysm location			0.1832
Anterior	32 (52%)	13 (21%)	
Posterior	9 (14%)	8 (13%)	
Multiple aneurysms	0	0	
Admission Hunt-Hess score			< 0.0001
1	28 (45%)	0	
2	4 (6%)	10 (16%)	
3	7 (11%)	3 (5%)	
4	2 (3%)	4 (7%)	
5	0	4 (7%)	

*MRS* Modified Rankin Scale, *DSA* digital subtraction angiography

ATP levels in the CSF of SAH patients were significantly higher compared with the control group (Con vs. SAH, 0.26 ± 0.28 µM vs. 4.16 ± 0.61 µM) (Fig. 1d). ATP levels in the SAH group were positively correlated with the H–H score (H–H 0–2 vs. H–H 3–5, 4.04 ± 0.49 µM vs. 4.41 ± 0.77 µM) (Fig. 1e), suggesting that ATP levels in the CSF of SAH patients were positively correlated with the severity of injury. Also, the findings showed that ATP levels in CSF also predicted the prognosis of SAH patients (MRS 0–2 vs. MRS 3–6, 4.02 ± 0.47 µM vs. 4.44 ± 0.78 µM) (Fig. 1f).

We performed receiver operating characteristic (ROC) curve analysis to determine the accuracy of USP30 level versus ATP level predictors in the CSF. In the CSF of patients in the control and SAH groups (Fig. 1g), the AUC of USP30 predictor was 0.5946, which did not have predictive accuracy, while the AUC of ATP predictor was 1, which could be considered as a perfect predictor. In the SAH group, the AUCs of USP30 and ATP predictors were 0.6446 and 0.6333 for the H-H score (Fig. 1h), respectively, with low accuracy, and 0.6214 and 0.6481 for the MRS score (Fig. 1i), respectively, with the same low accuracy.

### Hb Stimulation of Neurons Leads to Mitochondrial Damage and Apoptosis

**Fig. 2:**
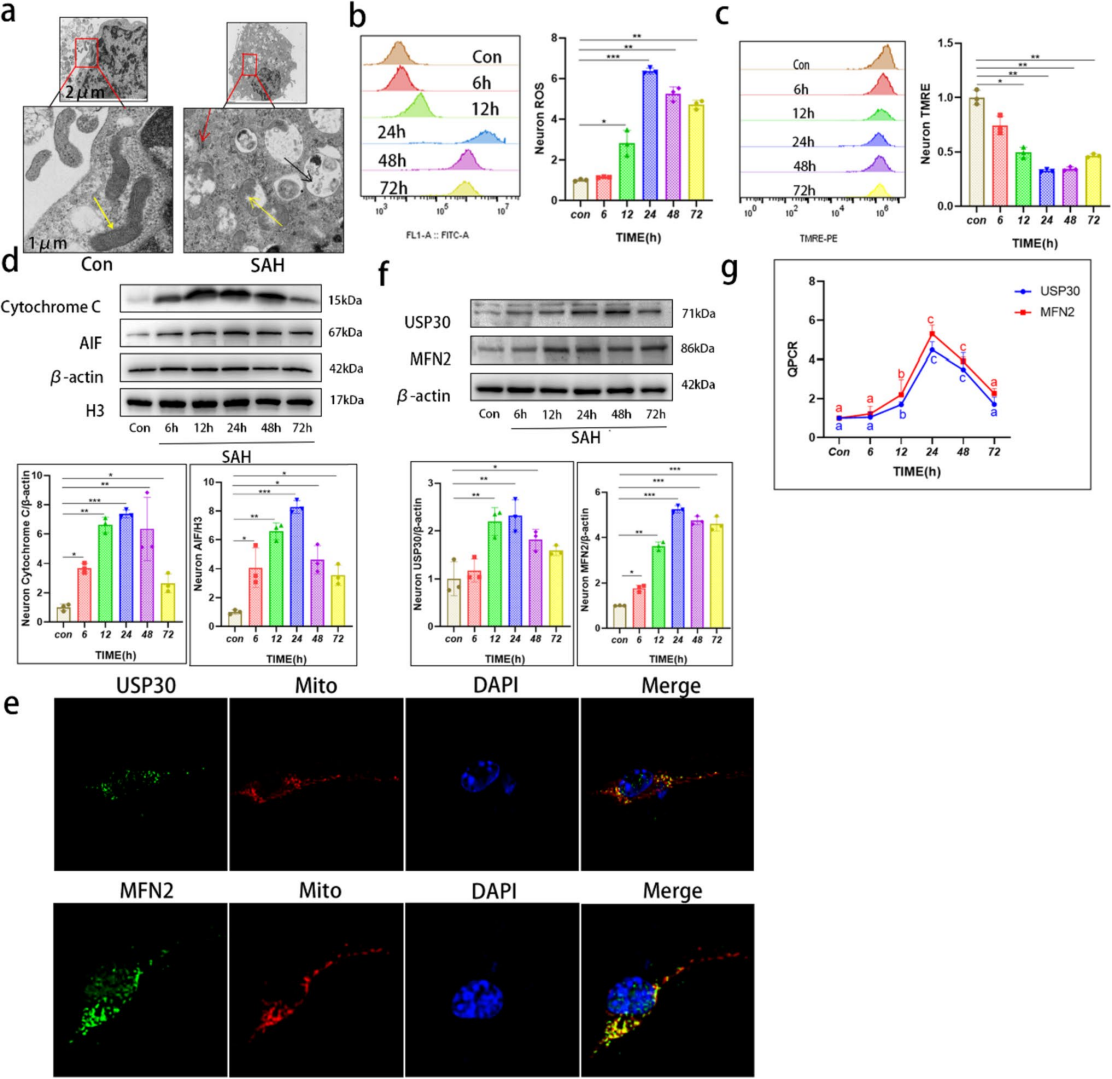
Increased USP30 expression after Hb stimulation of neurons is accompanied by apoptosis and mitochondrial damage. **a** Representative electron micrographs of neurons without Hb stimulation and after 24 h of Hb stimulation. Yellow arrows indicate mitochondria, red arrows indicate endoplasmic reticulum, and black arrows indicate autophagic vesicles; scale bars are marked in the images. **b, c** ROS fluorescent probe and mitochondrial membrane potential probe TMRE were incubated with Hb-stimulated neurons at 0, 6, 12, 24, 48, and 72 h for 30 min, and the number and fluorescence intensity of positive cells were detected by FC (*n* = 3, one-way ANOVA). **d** Protein expression levels of cytochrome C in the cytoplasm, AIF in the nucleus, and **f** USP30 and MFN2 in mitochondria after Hb stimulation for 0, 6, 12, 24, 48, and 72 h) (*n* = 3, one-way ANOVA). **e** Localization of USP30, MFN2, and mitochondria after 24 h of Hb stimulation. Bar = 20 µm. Green: USP30, MFN2; red: mitochondria; blue: DAPI. **g** The mRNA expression of USP30 and MFN2 after Hb stimulation for 0, 6, 12, 24, 48, and 72) (*n* = 3, one-way ANOVA). ns: not significant, *P < 0.05, **P < 0.01, and ***P < 0.001

The structure of neurons after SAH was abnormal, with sparse cell structure compared to normal cells by electron microscopy. Many mitochondria were damaged and necrotic, and the mitochondrial cristae were reduced or undetectable. A small number of autophagic vesicles containing various components, such as mitochondria and fragments of the endoplasmic reticulum, were seen wrapped by the bilayer membrane (Fig. 2a). A large amount of reactive oxygen species (ROS) are released from mitochondria along with the onset of SAH, reaching peak values at 24 h (Fig. 2b). In contrast, the mitochondrial membrane potential decreased continuously with a nadir at 24 h (Fig. 2c).

Cytochrome C and apoptosis-inducing factor (AIF) are located in the inner-outer membrane and membrane gap of mitochondria, respectively. When mitochondria are dysfunctional, cytochrome C enters the cytoplasm in large quantities, while AIF is transferred to the nucleus, causing DNA agglutination and breakage, both of which induce apoptosis [25]. The cytochrome C in the cytoplasm of neurons increased rapidly after SAH compared with the control group, reaching a peak at 24 h. The subsequent 48 h and 72 h decreased compared with 24 h but remained high (Fig. 2d). The AIF in the nucleus was significantly different from the control group at 6 h, peaked at 24 h, and decreased at 48 h and 72 h (Fig. 2d). SAH makes the neurons apoptotic in large numbers, and irreversible damage to mitochondria occurs.

### Increased USP30 Expression and MFN2 Accumulation on Mitochondria After Hb Stimulation of Neurons

We observed the intracellular distribution of USP30 and MFN2 using the IF assay, and the localization results showed that both were highly expressed in mitochondria after SAH (Fig. 2e). The expression of USP30 and MFN2 in the mitochondria various times after SAH was examined. The extracted proteins were measured using WB: USP30 started to increase at 6 h after SAH, reaching a maximum of 24 h. The subsequent 48 h and 72 h decreased, but still showed a substantial increase compared with the control group (Fig. 2f). MFN2 also started accumulating at 6 h after SAH and showed high expression at 24 h compared with the control group; the subsequent expression at 48 h and 72 h did not change significantly (Fig. 2f).

We then examined the mRNA expression of USP30 and MFN2 in the 6, 12, 24, 48, and 72 h for verification. The results were consistent with WB experiments and peaked at 24 h (Fig. 2g). Therefore, we chose 24 h as the observation time for subsequent experiments.

### Inhibition of USP30 After Hb Stimulation of Neurons Can Mediate Mitochondrial Protection and Attenuate Apoptosis

**Fig. 3:**
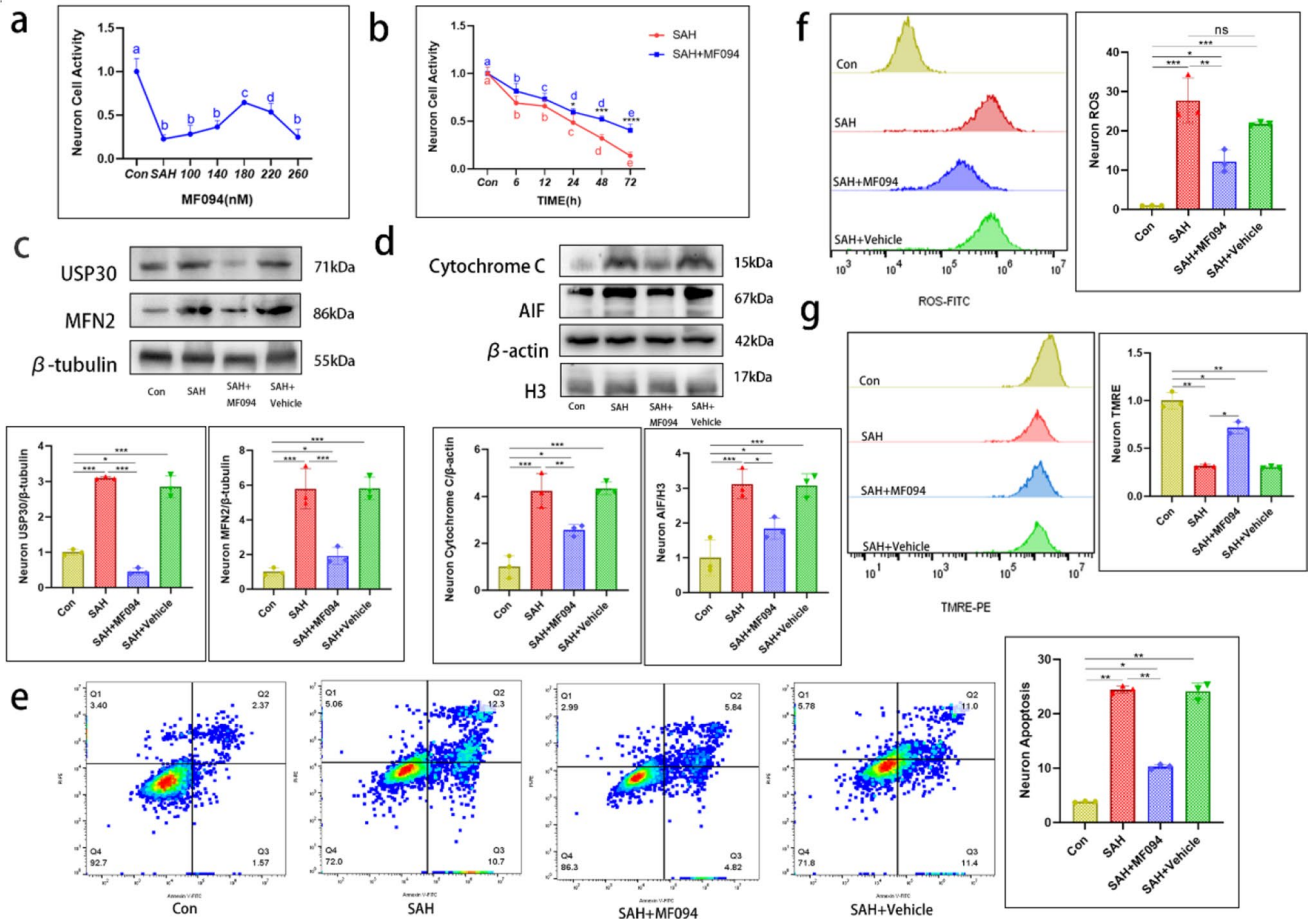
Inhibition of USP30 has a protective effect on neurons and mitochondria. **a** Viability of neurons in control and Hb-stimulated groups treated with various concentrations of MF094 (0 nM, 100 nM,140 nM, 180 nM, 220 nM, and 260 nM) for 24 h (*n* = 4, *t*-test). **b** Cell viability of neurons treated with MF094 at 180 nM concentration after Hb stimulation versus cells without MF094 treatment at 0, 6, 12, 24, 48, and 72 h (*n* = 6, *t*-test). **c, d** Expression levels of USP30, MFN2, cytochrome C, and AIF proteins in control, Hb-stimulated, MF094-treated, and null groups (*n* = 3, one-way ANOVA). **e** FC measurement of neuron apoptosis in control, Hb-stimulated, MF094-treated, and no-load groups (*n* = 3, one-way ANOVA). **f, g** FC measurement of ROS and mitochondrial membrane potential in control neurons, Hb-stimulated, MF094-treated, no-load groups, and fluorescence intensity calculation (*n* = 3, one-way ANOVA). ns: not significant, *P < 0.05, **P < 0.01, and ***P < 0.001

We set five concentration gradients of MF094 from 100 to 260 nM, added it to neurons after SAH, and cultured for 24 h, followed by CCK8 experiments. Neurons showed a maximum survival rate at 180 nM (64.50 ± 3.70%) (Fig. 3a). The survival rate of neurons after SAH showed a substantial increase from 24 to72 h after treatment with 180 nM MF094 (Fig. 3b). MF094 had a significant inhibitory effect on USP30 after SAH (SAH vs. SAH + MF094, 3.10 ± 0.04 vs 0.46 ± 0.10) (Fig. 3c). Correspondingly, MFN2 expression was significantly reduced (SAH vs. SAH + MF094, 5.79 ± 1.15 vs. 1.92 ± 0.49) (Fig. 3c). Therefore, we chose a drug concentration of 180 nM for subsequent experiments.

Neuronal apoptosis revealed that MF094 treatment significantly reduced cytochrome C in the cytoplasm (SAH vs. SAH + MF094, 4.25 ± 0.72 vs. 2.57 ± 0.24) and AIF expression in the nucleus (SAH vs. SAH + MF094, 3.12 ± 0.42 vs. 1.84 ± 0.31) (Fig. 3d). Annexin V/PI staining revealed that USP30 inhibition significantly reduced apoptosis in neurons after SAH (SAH vs. SAH + MF094, 24.47 ± 0.65% vs. 10.28 ± 0.41%) (Fig. 3e). We then examined the effect of USP30 inhibition on the alteration of ROS release and mitochondrial membrane potential after SAH. The MF094-treated neurons had a substantial reduction in ROS levels (SAH vs. SAH + MF094, 27.78 ± 5.69 vs. 12.23 ± 2.80) according to FC experiments (Fig. 3f), while the mitochondrial membrane potential was significantly increased (SAH vs. SAH + MF094, 31.54 ± 1.67% vs. 71.38 ± 6.14%) (Fig. 3g).

### Inhibition of USP30 Promotes Mitophagy

**Fig. 4:**
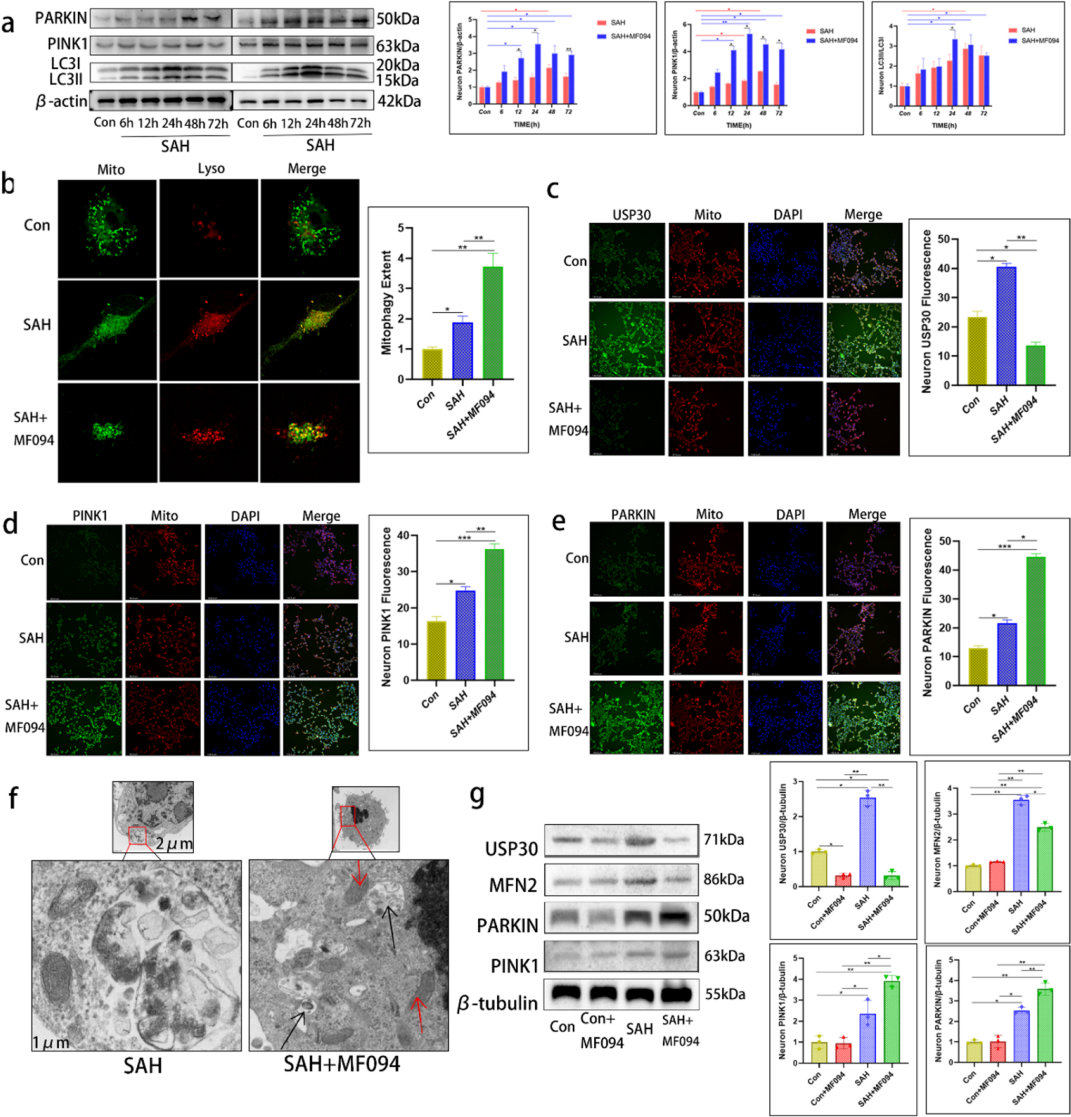
Inhibition of USP30 after Hb stimulation of neurons promotes early autophagy in damaged mitochondria. **a** Parkin, PINK1, and LC3 proteins were expressed in neurons without MF094 treatment and after MF094 treatment at 0, 6, 12, 24, 48, and 72 h after Hb stimulation (*n* = 3, two-way ANOVA). **b** Immunofluorescence staining to observe the co-localization of mitochondria with lysosomes in control, Hb-stimulated, and SAH + MF094 groups and to analyze the degree of localization. Bar = 20 µm. Green: mitochondria; red: lysosomes; yellow: fusion of mitochondria with lysosomes (*n* = 3, one-way ANOVA). **c–e** Fluorescence expression and fluorescence intensity analysis of USP30, PINK1, and Parkin protein in control, Hb-stimulated, and SAH + MF094 groups. Bar = 200 µm (*n* = 3, one-way ANOVA). **f** Representative electron micrographs of neurons untreated with MF094 and treated with MF094 for 24 h after Hb stimulation. Red arrows represent healthy mitochondria, and black arrows represent autophagic vesicles; scale bars are marked in the pictures. **g** USP30, Parkin, PINK1, and MFN2 protein expression in the control, Con + MF094, Hb-stimulated, and SAH + MF094 groups (*n* = 3, one-way ANOVA). ns: not significant, *P < 0.05 and **P < 0.01

We then investigated the pathways by which USP30 inhibition protects neurons and mitochondrial function. According to previous studies, mitophagy can clear damaged mitochondria, and USP30 is a key protease that regulates MFN2 ubiquitination during mitophagy. Therefore, we speculated that USP30 inhibition would be associated with promoting mitophagy. PTEN-induced putative kinase 1 (PINK1) and Parkin participate in mitophagy’s ubiquitin–proteasome pathway, while LC3 is the core receptor mediating autophagy onset. We measured the differential changes in PINK1, Parkin, and LC3 expression at various times after SAH and SAH + MF094 by WB experiments. A low degree of late mitophagy was generated in neurons after SAH, while USP30 inhibition led to increased mitophagy in neurons after SAH, which peaked at 24 h (Fig. 4a).

Lysosomes are responsible for the fusion and phagocytosis of autophagic vesicles to form autophagic lysosomes during mitophagy. We used fluorescence microscopy to observe the fusion of mitochondria and lysosomes. In the control neurons, the lysosomes were distributed around the mitochondria, and no fusion was observed; in the SAH group, the lysosomes partially engulfed and fused with the damaged mitochondria. With SAH + MF094 group, the lysosomes engulfed and fused almost all damaged mitochondria (Fig. 4b). The IF results also showed that the expression of mitophagy-related proteins was significantly increased in neurons treated with MF094 for 24 h after SAH while inhibiting USP30 expression (Fig. 4c–e).

### Inhibition of USP30 Only Promotes Clearance of Damaged Mitochondria

Next, we studied the effects of inhibiting USP30 on the intracellular mitochondria, which remain structurally and functionally intact, while removing damaged mitochondria. Using electron microscopy, we observed many autophagic vesicles in the cytoplasm of the cells after SAH with the inhibitor MF094. Many structurally intact mitochondria were also observed with their overall fullness and clear bilayer membrane structure, and no obvious breakage of mitochondrial cristae was seen to reduce their disappearance. Undamaged mitochondria did not show autophagy (Fig. 4f).

We then examined the changes in mitophagy after USP30 inhibition in normal neurons. With USP30 inhibition, PINK1, Parkin, and MFN2 proteins in the cells of the Con + MF094 group did not undergo significant changes compared to the control group. In contrast, the changes in protein expression in the SAH and SAH + MF094 groups were consistent with the previous experiments (Fig. 4g). Therefore, we speculated that MF094 reduces the USP30 of cells in the normal state but does not affect their mitophagy, and the mitochondrial state in normal cells is unchanged.

### Inhibition of USP30 Promotes Ubiquitination of MFN2 by Parkin

**Fig. 5:**
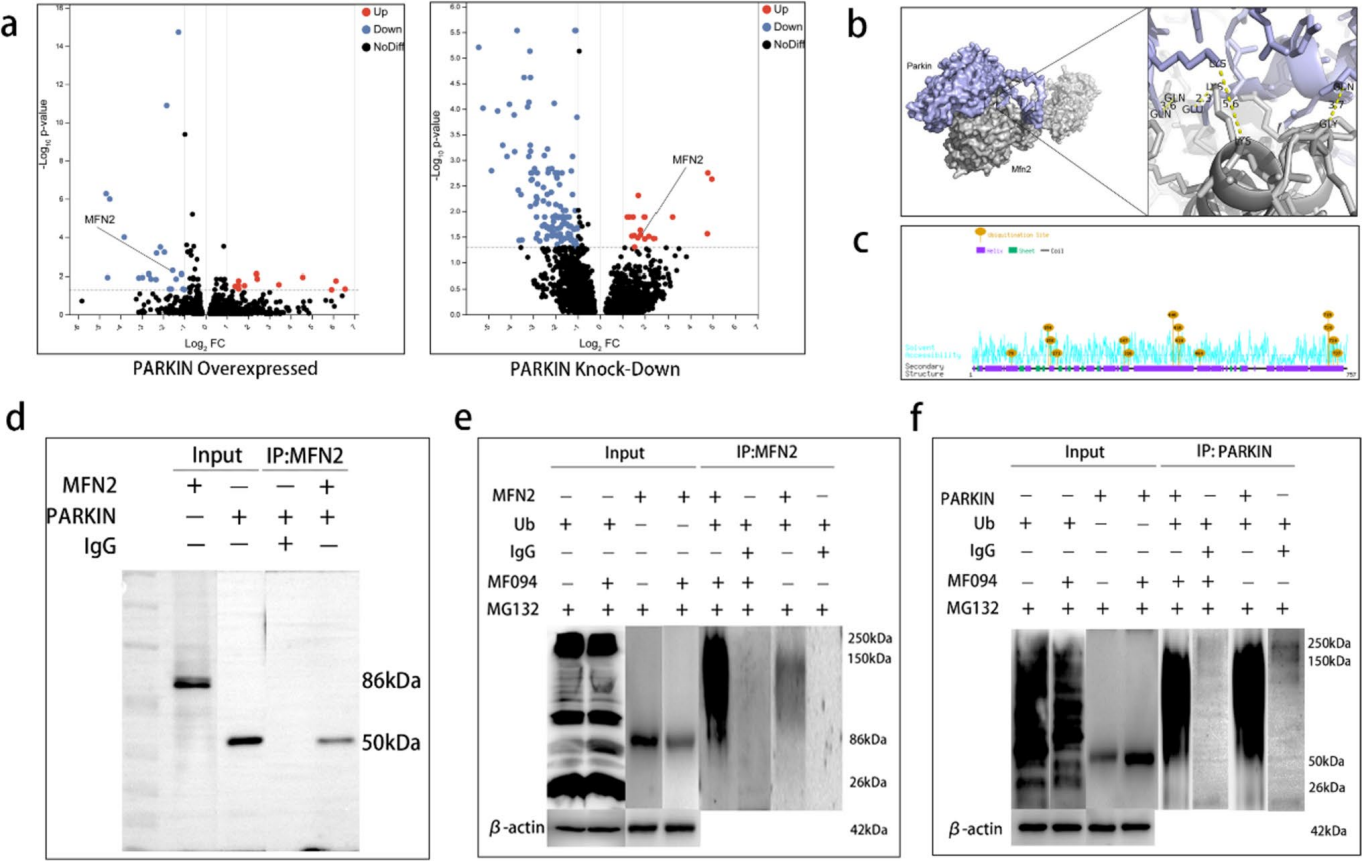
Inhibition of USP30 promotes ubiquitination of MFN2 by Parkin. **a** Change in MFN2 expression upon Parkin protein overexpression and knockdown (Parkin overexpressed fold-change: − 1.316, *P* = 0.01942) (Parkin knockdown fold-change: 1.828, *P* = 0.02986). **b** Molecular docking model of Parkin and MFN2 (distance less than 5 Å is indicated as having significant interaction force). **c** Motif analysis of the binding sites of Parkin and MFN2. **d** Immunoprecipitation assay to detect the binding of Parkin to MFN2. **e** The extent of MFN2 protein ubiquitination in neurons after Hb stimulation without MF094 treatment vs. after MF094 treatment. **f** Ubiquitination of Parkin protein in neurons after Hb stimulation without MF094 treatment versus after MF094 treatment

Parkin regulates the ubiquitination of MFN2, a critical step in mitophagy. Parkin is recruited to mitochondria, binds to phosphorylated MFN2, and ubiquitinates it, thereby degrading it [26]. First, we examined the differentially expressed genes. A volcano plot revealed that MFN2 was significantly downregulated in Parkin-overexpressing mice compared to the control group (fold-change − 1.316, *P* = 0.01942). MFN2 was significantly upregulated in Parkin knockout mice compared to the control group (fold-change 1.828, *P* = 0.02986) (Fig. 5a). We then predicted the binding pattern of Parkin to MFN2 using molecular docking. There were significant hydrophobic interaction forces between MFN2-LYS-316 and Parkin-LYS-151, MFN2-LYS-316 and Parkin-LYS-309, and MFN2-GLN-339 and Parkin-GLN-155. The molecular docking results indicate significant protein Parkin and MFN2 interaction forces and the possibility of interaction in space (Fig. 5b). Finally, Motif results showed the highest confidence scores for lysine at positions 158 and 406 of MFN2, indicating that LYS-158 and LYS-406 may be the ubiquitination sites of Parkin (Fig. 5c).

USP30 reverses the ubiquitination process by removing the ubiquitin tag placed on MFN2 by Parkin. After precipitation of Parkin protein using an antibody, Parkin binding to MFN2 was verified using WB (Fig. 5d). Ubiquitination experiments showed that the expression of ubiquitin protein on MFN2 does not change, however, ubiquitination of MFN2 was enhanced after inhibition of USP30 (Fig. 5e). However, Parkin is itself ubiquitinated; therefore, we asked whether USP30 also inhibits this process. We found that ubiquitin protein expression on Parkin and Parkin ubiquitination were unchanged after USP30 inhibition (Fig. 5f).

### Inhibition of USP30 Reduced Extracellular ATP Levels

**Fig. 6:**
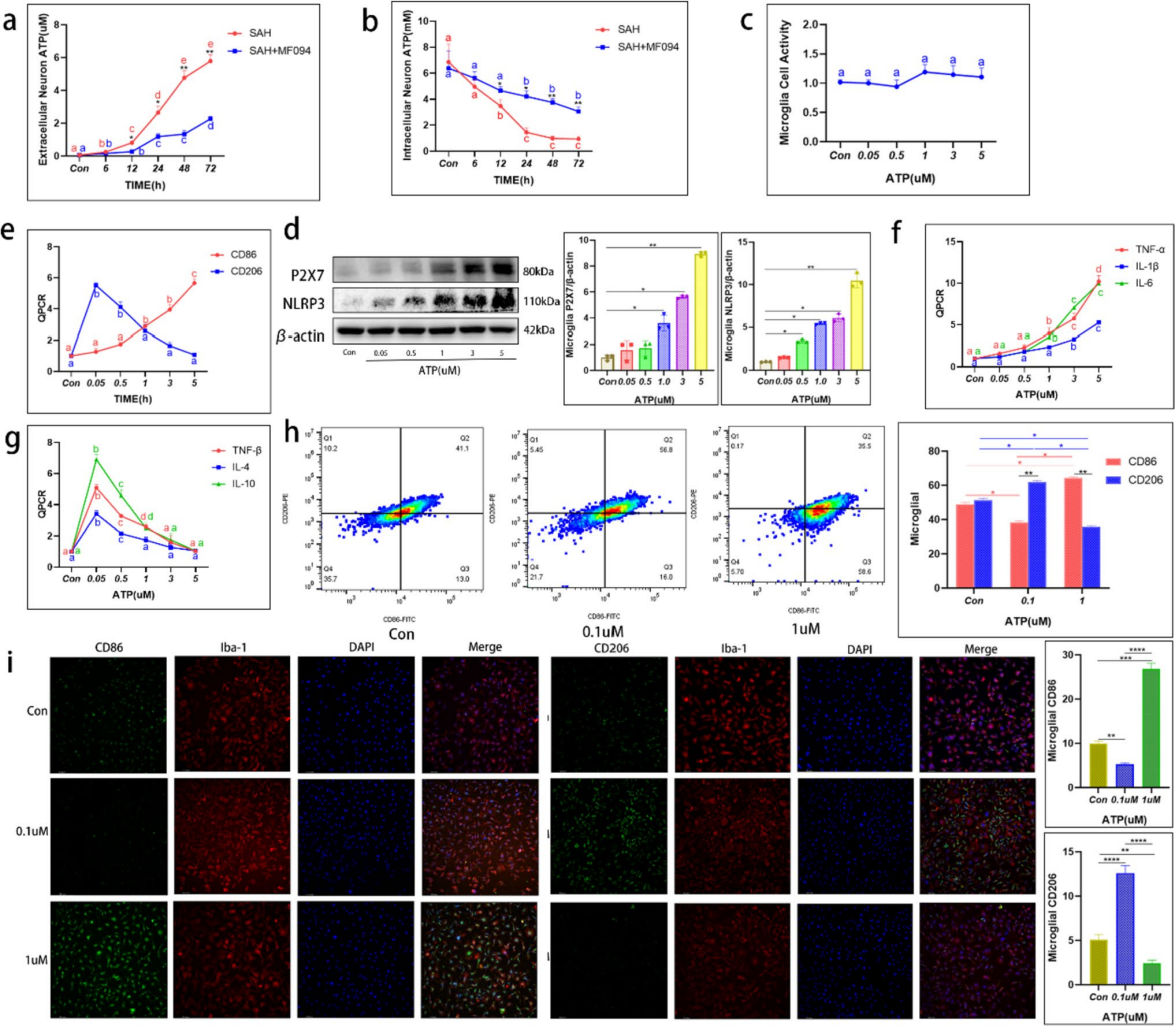
Activation of microglia by the ATP-P2X7 pathway triggers an inflammatory response. **a** Extracellular ATP levels in untreated and MF094-treated neurons after Hb stimulation at 0, 6, 12, 24, 48, and 72 h (*n* = 6, two-way ANOVA). **b** ATP levels at 0, 6, 12, 24, 48, and 72 h in untreated and MF094-treated neurons after Hb stimulation (*n* = 6, two-way ANOVA). **c** Cell viability assay of microglia treated with different concentrations of ATP (0 µM, 0.05 µM, 0.5 µM, 1 µM, 3 µM, and 5 µM) for 24 h (*n* = 6, *t*-test). **d** Protein expression of P2X7 and NLRP3 after 24 h treatment with different concentrations of ATP (0 µM, 0.05 µM, 0.5 µM, 1 µM, 3 µM, and 5 µM) in microglia (*n* = 3, one-way ANOVA). **e** mRNA expression of CD86 and CD206 in microglia treated with different concentrations of ATP (0 µM, 0.05 µM, 0.5 µM, 1 µM, 3 µM, and 5 µM) for 24 h (*n* = 3, one-way ANOVA). **f** mRNA expression of TNF-α, IL-1β, and IL-6 in microglia treated with different concentrations of ATP (0 µM, 0.05 µM, 0.5 µM, 1 µM, 3 µM, and 5 µM) for 24 h (*n* = 3, one-way ANOVA). **g** mRNA expression of TNF-β, IL-4, and IL-10 in microglia treated with different concentrations of ATP (0 µM, 0.05 µM, 0.5 µM, 1 µM, 3 µM, and 5 µM) for 24 h (*n* = 3, one-way ANOVA). **h** The polarization direction of microglia after 24-h treatment with different concentrations of ATP (0 µM, 0.1 µM, and 1 µM) was detected by FC (*n* = 3, two-way ANOVA). **i** Fluorescence expression and fluorescence intensity analysis of CD86 and CD206 on the surface of microglia after 24-h treatment with different concentrations of ATP (0 µM, 0.1 µM, and 1 µM). Bar = 200 µm. ns: not significant, *P < 0.05, **P < 0.01, ***P < 0.001, and ****P < 0.0001

We measured the extracellular ATP levels of neurons, which were cultured in 5 mL of medium. Extracellular ATP levels increased rapidly after SAH at 12–48 h. While the extracellular ATP levels were substantially reduced after USP30 inhibition compared to the SAH group (Fig. 6a).

We then considered whether MF094 reduces the extracellular ATP level by inhibiting intracellular ATP production. We measured intracellular ATP levels and found that they decreased after SAH and were lower than the normal values (1–10 mM), while the intracellular ATP was significantly elevated in MF094-treated cells compared with the SAH group and was always within the normal range (Fig. 6b).

### Activation of P2X7 Receptors and NLRP3-Type Inflammatory Vesicles on Microglia by ATP Promotes the Release of Inflammatory Factors

Extracellular ATP can activate inflammatory vesicles such as NLRP3 and release inflammatory factors through the P2X7 receptor pathway. We generated ATP solutions with concentrations from 50 nM to 6 µM added to microglia based on the extracellular ATP levels of neurons after SAH was measured previously. The survival of microglia increased slightly but not significantly with increasing extracellular ATP concentration (Fig. 6c).

Next, the activation of P2X7 and NLRP3 on microglia after 24 h of ATP stimulation at different concentrations was examined using WB. P2X7 and NLRP3 expression profiles were positively correlated with ATP concentration (Fig. 6d). The release of inflammatory factors was observed using qPCR experiments and showed that with the increase of ATP concentration, the polarized microglia shifted from M2 to M1 type (Fig. 6e). Pro-inflammatory factors such as IL-1β, TNF-α, and IL-6 increased accordingly (Fig. 6f); IL-4, IL-10, and TNF-β and other anti-inflammatory factors decreased with the increase of ATP level (Fig. 6g).

Finally, microglia were stimulated with 100 nM and 1 µM concentrations of ATP. IF and FC revealed that the expression of CD206 on the surface of microglia increased in the low ATP environment, converting toward the M2 type. The expression of the marker CD86 on the surface of microglia was predominant in the high ATP environment, predicting a conversion toward the M1 type (Fig. 6h, i).

### USP30 Inhibition Improves SAH Outcomes in Mice, Increasing Neuronal Survival and Reducing Brain Edema

**Fig. 7:**
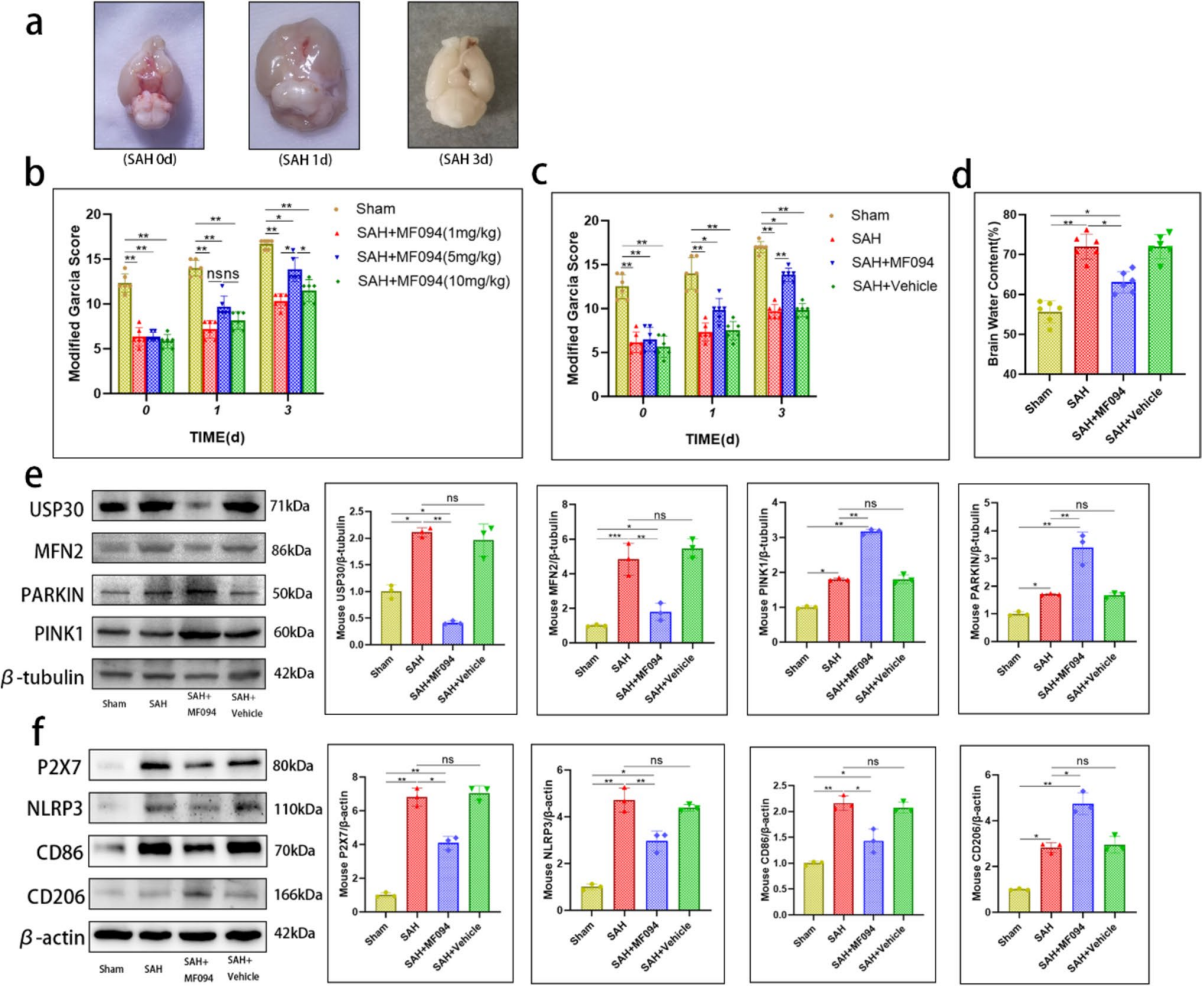
Inhibition of USP30 reduces brain injury and improves outcomes in mice after SAH. **a** The extent of brain injury in mice at 0 d, 1 d, and 3 d after SAH. **b** Outcomes were assessed using the modified Garcia score after 3 days of treatment with different concentrations of MF094 after SAH (*n* = 6, two-way ANOVA). **c** Modified Garcia score of mice in the sham-operated group, SAH group, SAH + MF094 group, and SAH + Vehicle group at 3 days (*n* = 6, two-way ANOVA). **d** Brain water content of mice in the sham, SAH, SAH + MF094, and SAH + Vehicle groups after 3 days (*n* = 6, one-way ANOVA). **e** Expression of USP30, MFN2, Parkin, and PINK1 in the brain tissue of mice in sham, SAH, SAH + MF094, and SAH + Vehicle groups after 3 d (*n* = 3, one-way ANOVA). **f** Expression of P2X7, NLRP3, CD86, and CD206 in brain tissues of mice in the sham, SAH, SAH + MF094, and SAH + Vehicle groups after 3 d (*n* = 3, one-way ANOVA). ns: not significant, *P < 0.05 and **P < 0.01

Using animal experiments, we observed the effect of USP30 inhibition on injury and outcomes after SAH. The recovery of mice after SAH at 0, 1, and 3 days was assessed using the modified Garcia score. MF094 had the best therapeutic effect at 5 mg/kg (*n* = 6, Fig. 7b). Therefore, we used 5 mg/kg in subsequent experiments. A slight improvement was observed in the SAH + MF094 group mice one day after SAH compared to the SAH group (SAH vs. SAH + MF094, 7.33 ± 1.03 vs. 9.83 ± 1.33). There was a substantial improvement in outcomes at 3 d compared to the SAH group (SAH vs. SAH + MF094, 9.67 ± 0.82 vs. 13.83 ± 0.75) (*n* = 6, Fig. 7c). A brain water content assay demonstrated that USP30 inhibition significantly reduced brain edema in mice after SAH (SAH vs. SAH + MF094, 72.02 ± 3.13% vs. 63.10 ± 2.65%) (*n* = 6, Fig. 7d).

**Fig. 8:**
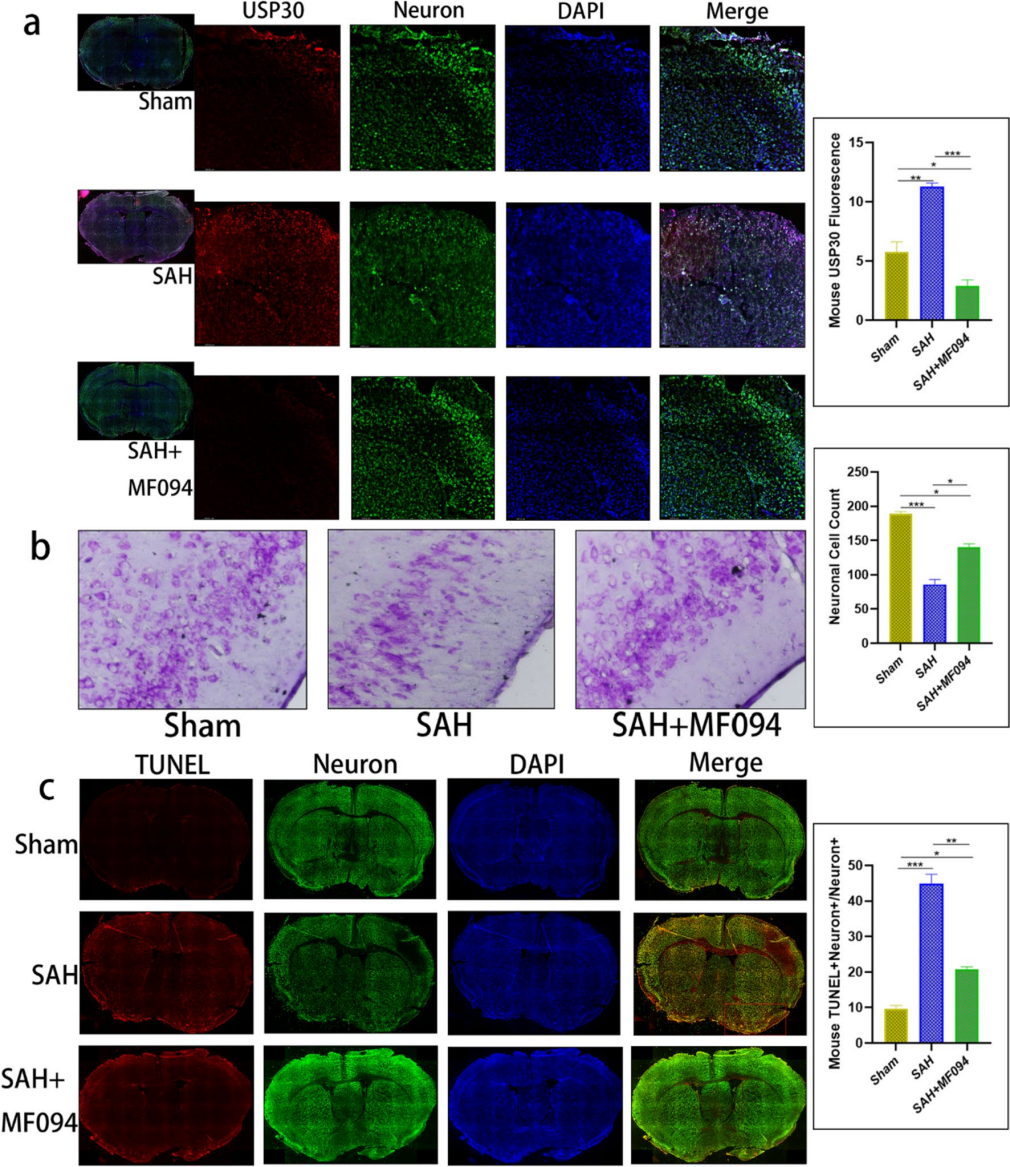
Immunofluorescence staining of mouse brain tissues. **a** Immunofluorescence staining and fluorescence intensity analysis of USP30 protein in brain tissues of mice in the sham group, SAH, and SAH + MF094 groups after 3d. Bar = 200 µm (*n* = 3, one-way ANOVA). ns: not significant, *P < 0.05, **P < 0.01, and ***P < 0.001. **b** Nissl staining and neuron counting analysis of brain tissue after 3 d in the sham, SAH, and SAH + MF094 groups. Bar = 200 µm (*n* = 3, one-way ANOVA). **c** TUNEL staining and neuronal apoptosis analysis of mice in the sham, SAH, and SAH + MF094 groups after 3 d. Apoptotic tissues are shown in red boxes. Bar = 1 mm (*n* = 3, one-way ANOVA)

Brain tissue proteins were extracted, and protein expression was measured; the WB results were consistent with the in vitro experiments (Fig. 7e, f). Brain tissue IF also fit the change of USP30 expression in cells after MF094 treatment (*n* = 3, Fig. 8a). Using Nissl staining, we found that USP30 inhibition increased neuronal survival after SAH (SAH vs. SAH + MF094, 86.33 ± 3.51 vs. 142.70 ± 7.02) (*n* = 3, Fig. 8b). The TUNEL + NeuN + ratio of SAH mice treated with MF094 was significantly lower than that of the SAH group (SAH vs. SAH + MF094, 44.80 ± 2.72% vs. 20.73 ± 0.74%), suggesting that MF094 reduces neuronal apoptosis in mice after SAH (*n* = 3, Fig. 8c).

## Discussion

Our main finding is that USP30 inhibition mediates protection from SAH-induced mitochondrial damage. To verify this effect, we used bovine hemoglobin to create an in vitro model of SAH and an in vivo mouse model by an intravascular puncture to elucidate the essential involvement of USP30. USP30 inhibition clears damaged mitochondria by promoting mitophagy and rescues neuronal apoptosis and extracellular high ATP environment after SAH. USP30 removed Parkin-mediated MFN2 ubiquitination during mitophagy, causing MFN2 accumulation, which resulted in damaged mitochondria being able to continue to fuse and causing sustained cellular damage. USP30 inhibition reversed this process. With the removal of damaged mitochondria, the extracellular high ATP environment was altered, inhibiting the activation of microglia toward the pro-inflammatory phenotype and avoiding inflammatory damage to the nervous system.

USP30 is widely distributed on the OMM in all human cells; in the present study, USP30 expression increased rapidly after SAH onset, while mitochondrial integrity was irreversibly disrupted. Bingol et al. proposed that USP30 removes ubiquitin attached to damaged mitochondria via Parkin and blocks Parkin-driven mitochondrial phagocytosis. In contrast, reducing the activity of USP30 enhances the degradation of damaged mitochondria in neurons. USP30 knockdown rescued the defective mitochondrial phagocytosis caused by pathogenic mutations in Parkin and improved mitochondrial integrity in PINK1 or Parkin-deficient Drosophila [27]. Based on this background, we selected USP30 as a target for clearing damaged mitochondria after SAH. Previous studies on the mechanism of USP30 action in mitophagy have focused on Parkinson’s disease [28], with minimal attention on SAH. In the present study, we focused on the effect of USP30 inhibition on mitophagy after SAH and provided insights into the mechanism and compensation for apoptosis and inflammatory damage after SAH.

Neuronal apoptosis critically affects outcomes in SAH patients. Our study found that damaged mitochondria release large amounts of ROS and decrease membrane potential. Mladenov et al. proposed that ROS are a significant source of oxidative stress and that the accumulation of ROS is a consequence of stroke and reperfusion, leading to inflammatory and immune responses that can further damage neurovascular units [29]. Sun et al. found that a decreased mitochondrial membrane potential triggers mitochondrial enzyme activity, disrupting the normal functioning of the respiratory chain system and affecting ATP synthesis [30]. These are both caused by apoptosis and, in turn, promote apoptosis. In our study, USP30 inhibition reduced apoptosis for unknown reasons. Because USP30 inhibition positively affects ROS clearance and restoration of mitochondrial membrane potential, we speculate that the phenomenon may be related to the clearance of damaged mitochondria. Liang et al. reported that USP30 inhibition reduced apoptosis by regulating the BAX/BAK pathway [31, 32]; we will follow up on the specific mechanism.

**Fig. 9:**
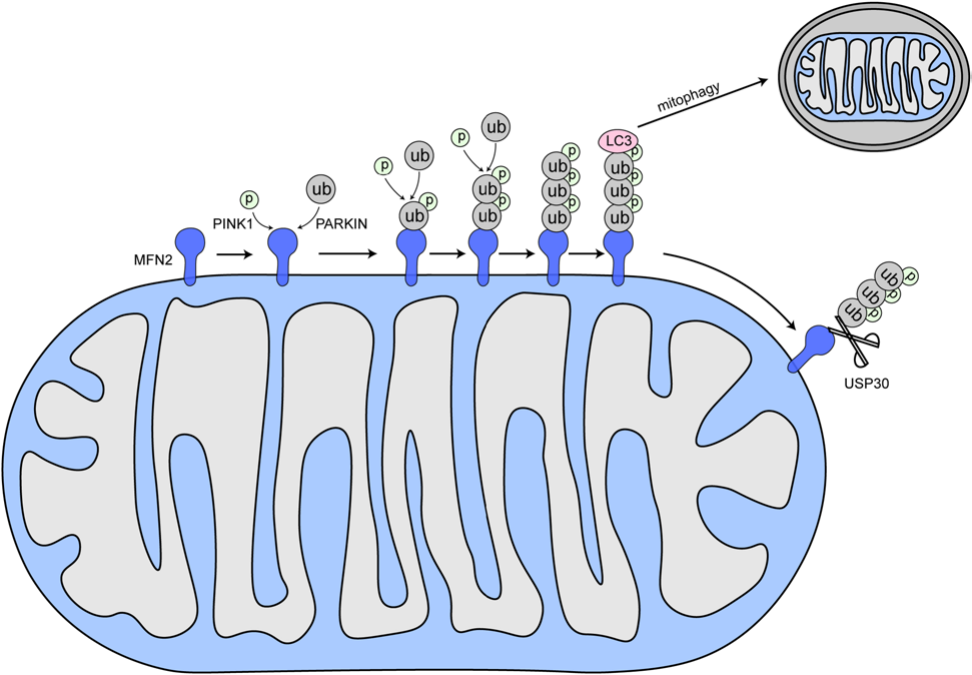
After SAH, the PINK1/Parkin pathway is activated and MFN2 is phosphorylated and ubiquitinated, respectively, leading to degradation of MFN2, which inhibits normal fusion of damaged mitochondria and promotes mitophagy. USP30 can counteract mitophagy from occurring by removing the ubiquitination of MFN2

The ubiquitin–proteasome system plays an important role in mitophagy [33]. When mitochondria are damaged, threonine at position 111 and serine at position 442 of MFN2 localized to the OMM is phosphorylated by PINK1 and recruited to the mitochondria, which are then bound to Parkin and modified by ubiquitination [34]. Increased degradation of ubiquitinated MFN2 via the proteasome pathway inhibits mitochondrial fusion, resulting in damaged mitochondria breaking into granules that are subsequently encapsulated by autophagic vesicles to form autophagosomes that fuse with lysosomes to degrade and produce mitophagy [26, 35] (Fig. 9). Quarato et al. proposed that the E3 ubiquitin ligase function of the Parkin protein ubiquitinates OMM substrates and recruits the ubiquitin–proteasome system and a small class of cytoplasmic protein autophagy receptors to damaged mitochondria [36]. These receptors contain the LC3 interaction region structural domain, which recruits members of the autophagy-associated 8 family, ultimately forming autophagic vesicles around mitochondrial fragments and fusion with lysosomes [37].

The ubiquitination of MFN2 by Parkin proteins is essential to initiating the mitophagy process [38]. Henriques et al. showed that USP30 inhibition increased the ubiquitination of MFN2 and led to the extension of the mitochondrial network, while USP30 also depended on MFN2 to regulate mitochondrial morphology [39]. However, contrary to our findings, Yue et al. found that USP30 mediated non-degradative ubiquitination of MFN2 [9]. A possible explanation is that Yue et al. examined MFN2 changes by modulating USP30 in normal uninjured cells, whereas we focused on studying MFN2 changes in neurons that underwent apoptosis after SAH. The difference between the two has been elucidated in the above study.

The process of activation of P2X7 receptors on the microglia surface to produce NLRP3-type inflammatory vesicles in a high ATP environment has been reported [40–42]. Schädlich et al. pointed out that P2X7 activation is a potent trigger for NLRP3 inflammatory vesicle formation in macrophages and microglia, leading to the release of IL-1β, which is a potent driver of informatization after stroke [40]. However, deciphering the complex role of P2X7 in the interrelated processes of post-stroke infection, blood–brain barrier disruption, and neuronal recovery remains challenging. Tao et al. found that microglia phagocytosis was progressively impaired by continuous BzATP exposure and P2X7 activation [14]. In recent years, several studies found that activation of P2X7 can lead to impaired microglia phagocytosis in fibrillar Aβ [43].However, the precise mechanism of action is unclear; here, we only elaborated on the effect of inflammatory responses on microglia via the ATP-P2X7 pathway after SAH. We verified the inflammatory damage caused to the nervous system after ATP stimulation and found that USP30 inhibition reduced the high ATP environment in which microglia were exposed.

Our study provides insights into the clearance of damaged mitochondria after SAH; however, the present study has some limitations. First, we wanted to clear the damaged mitochondria by mitophagy, and although its possibility has been demonstrated in other studies, we did not directly demonstrate it in the present study. The idea of explaining the clearance of damaged mitochondria by only inhibiting USP30 to observe ROS production, restoration of mitochondrial membrane potential, and morphological changes of mitochondria under electron microscopy remains somewhat unlikely. Second, our study found that ATP was transferred from intracellular to extracellular after SAH, resulting in elevated extracellular ATP levels; in fact, a large amount of blood was released after SAH, which could potentially be the primary source of ATP. The difference between the two sources was not distinguished in our experiments. Finally, a large amount of sample data and long-term follow-up observations are needed regarding the prognostic impact of high USP30 levels after SAH and high ATP concentrations in CSF patients; we will conduct these experiments in future studies. Until the potential mechanism of USP30 for mitophagy is clear, its inhibition therapy may be limited in clinical application.

## Conclusion

Mitochondrial damage in neurons after SAH leads to apoptosis and a high extracellular ATP environment, and we hoped to reverse this phenomenon by removing damaged mitochondria through mitophagy. In contrast, elevated USP30 after SAH inhibited mitophagy. High ATP levels in the CSF of patients lead to inflammatory injury. Therefore, we inhibited USP30 and promoted ubiquitination of MFN2 by Parkin to degrade MFN2, which accelerated mitophagy. Ultimately, apoptosis and extracellular ATP levels were reduced after SAH.

## Data Availability

The data that support the findings of this study are available from the corresponding author upon reasonable request.
