# Correction to: Inhibition of USP30 Promotes Mitophagy by Regulating Ubiquitination of MFN2 by Parkin to Attenuate Early Brain Injury After SAH

**DOI:** 10.1007/s12975-024-01245-w

**Published:** 2024-04-18

**Authors:** Yang Liu, Chenbei Yao, Bin Sheng, Simin Zhi, Xiangxin Chen, Pengfei Ding, Jiatong Zhang, Zhennan Tao, Wei Li, Zong Zhuang, Jiannan Mao, Zheng Peng, Huiying Yan, Wei Jin

**Affiliations:** 1 Department of Neurosurgery, Nanjing Drum Tower Hospital Clinical College of Nanjing University of Chinese Medicine, Nanjing, 210008 Jiangsu China; 2 Department of Neurosurgery, Affiliated Hospital of Medical School, Nanjing Drum Tower Hospital, Nanjing University, Nanjing, 210008 Jiangsu China; 3 Department of Neurosurgery, Nanjing Drum Tower Hospital Clinical College of Nanjing Medical University, Nanjing, 210008 Jiangsu China

**Correction to: Translational Stroke Research**

10.1007/s12975-023-01228-3

In the “AIF”group in Figure 3d, the old version of the protein strips were of lower quality and we used new protein strips that were more recognizable.

Fig.3d 

Modified Fig.3d 

In the “SAH”group in Figure 4b, the new version has a more representative picture.

Fig.4b 

Modified Fig.4b 

In the “Input”group in Fig. 5e, we concluded that ubiquitin protein expression was lower in neuronal cells after SAH in the absence of USP30 inhibition. However, after reviewing a large amount of literature, it was found that USP30 only removes the attachment of MFN2 to ubiquitin proteins and does not mediate a decrease in ubiquitin protein expression. The expression of ubiquitin protein will not be changed whether USP30 is inhibited or not. Previous studies may have considered that unskilled experimental techniques or improper protein preservation caused the degradation of ubiquitin protein, and thus gave wrong experimental results. After several repetitions of the experiment, we arrived at the correct results.

Fig. 5e 

Modified Fig. 5e 

Mischaracterization:

Ubiquitination experiments showed that ubiquitin protein expression on MFN2 was reduced, and ubiquitination of MFN2 was enhanced after USP30 inhibition (Fig. 5e).

Modified description:

Ubiquitination experiments showed that the expression of ubiquitin protein on MFN2 does not change, however, ubiquitination of MFN2 was enhanced after inhibition of USP30 (Fig. 5e).

The original article has been corrected.

