# Correction to: Inhibition of USP30 Promotes Mitophagy by Regulating Ubiquitination of MFN2 by Parkin to Attenuate Early Brain Injury after SAH

**DOI:** 10.1007/s12975-026-01418-9

**Published:** 2026-02-17

**Authors:** Yang Liu, Chenbei Yao, Bin Sheng, Simin Zhi, Xiangxin Chen, Pengfei Ding, Jiatong Zhang, Zhennan Tao, Wei Li, Zong Zhuang, Jiannan Mao, Zheng Peng, Huiying Yan, Wei Jin

**Affiliations:** 1 Department of Neurosurgery, Nanjing Drum Tower Hospital Clinical College of Nanjing University of Chinese Medicine, Nanjing, Jiangsu 210008 China; 2 Department of Neurosurgery, Affiliated Hospital of Medical School, Nanjing Drum Tower Hospital, Nanjing University, Nanjing, Jiangsu 210008 China; 3 Department of Neurosurgery, Nanjing Drum Tower Hospital Clinical College of Nanjing Medical University, Nanjing, Jiangsu 210008 China

**Correction to: Translational Stroke Research (2025) 16:448–466.**

10.1007/s12975-023-01228-3.

The online version of the original article can be found at.

10.1007/s12975-023-01228-3.

Following multiple careful reviews of the manuscript, we have identified an error in Fig. 8.b. This image was inadvertently taken from experimental results of another concurrent research project and was mistakenly included in place of the correct figure during the preparation of the manuscript. In the interest of scientific accuracy and rigor, we have provided the correct experiment results.

**Figure 1:**
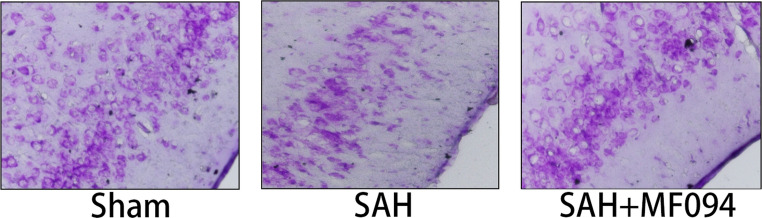
Fig. 8b

Modified Fig. 8b.


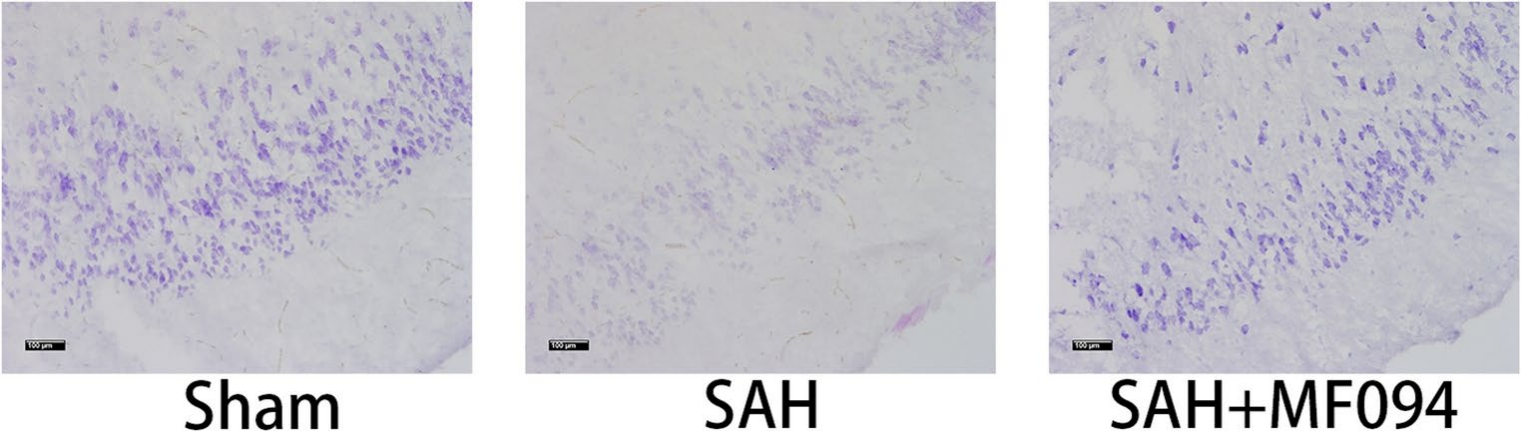


Modified Fig. 8.


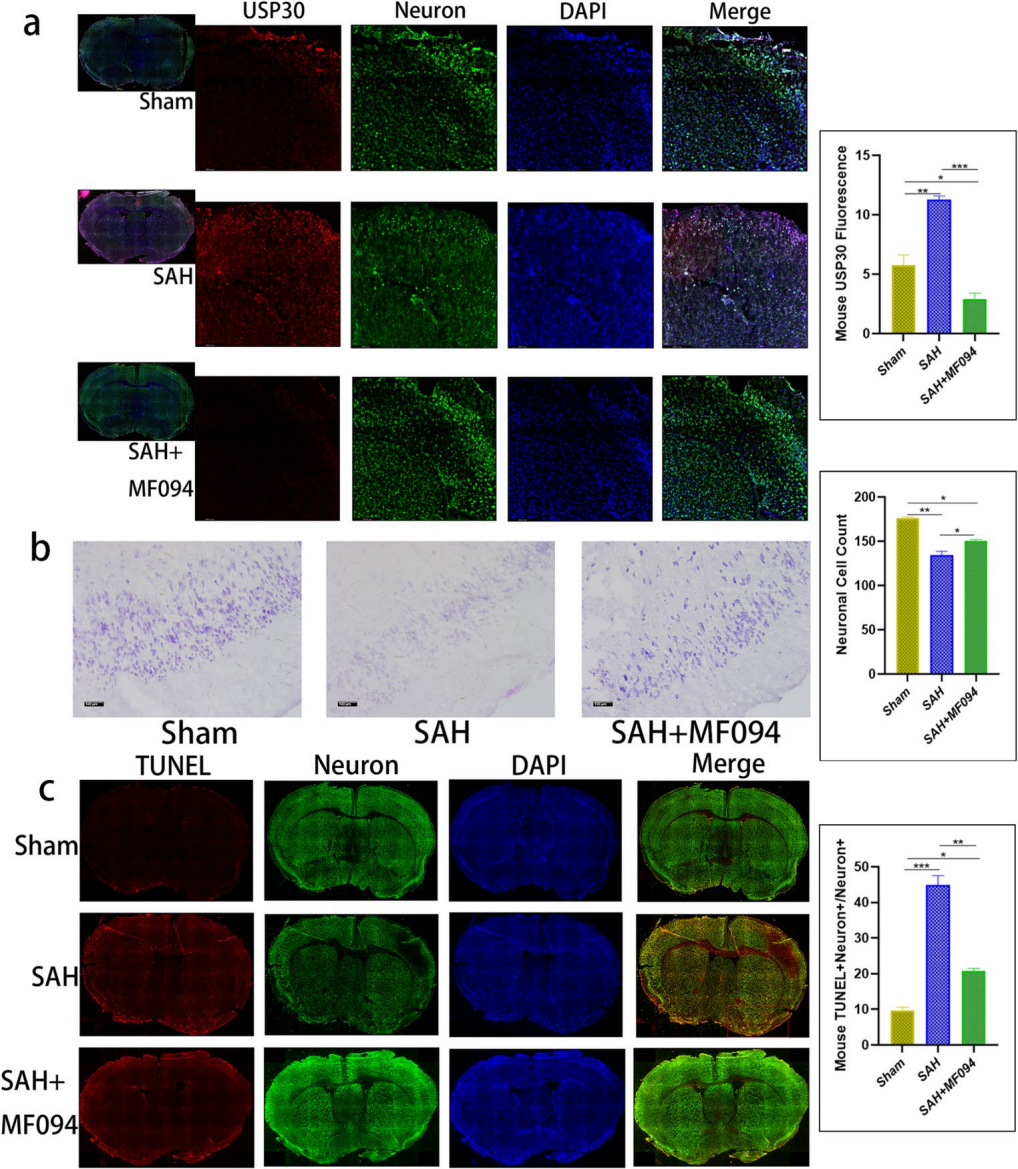


**Figure 8** Immunofluorescence staining of mouse brain tissues. **a** Immunofluorescence staining and fluorescence intensity analysis of USP30 protein in brain tissues of mice in the sham group, SAH, and SAH+MF094 groups after 3d.bar=200 μm (*n* = 3, one-way ANOVA). **b** Nissler staining and neuron counting analysis of brain tissue after 3d in sham, SAH, and SAH+MF094 groups.bar=100 μm (*n* = 3, one-way ANOVA). **c** TUNEL staining and neuronal apoptosis analysis of mice in sham, SAH, and SAH+MF094 groups after 3d. Apoptotic tissues are shown in red boxes.bar=1 mm (*n* = 3, one-way ANOVA).ns: not significant, *P < 0.05, **P < 0.01,***P < 0.001.

